# Implications of Social Competence among Thirty-Month-Old Toddlers: A Theory of Mind Perspective

**DOI:** 10.2188/jea.JE20090173

**Published:** 2010-03-05

**Authors:** Emiko Tanaka, Etsuko Tomisaki, Ryoji Shinohara, Yuka Sugisawa, Lian Tong, Taeko Watanabe, Yoko Onda, Yuri Kawashima, Maki Hirano, Yukiko Mochizuki, Kentaro Morita, Amarsanaa Gan-Yadam, Yuko Yato, Noriko Yamakawa, Shoji Itakura, Tamiko Ogura, Aya Kutsuki, Misa Kuroki, Tokie Anme, Japan Children’s Study Group

**Affiliations:** 1 Research Institute of Science and Technology for Society, Japan Science and Technology Agency, Tokyo, Japan; 2 Graduate School of Comprehensive Human Sciences, University of Tsukuba, Tsukuba, Ibaraki, Japan; 3 College of Letters, Ritsumeikan University, Kyoto, Japan; 4 Clinical Research Institute, Mie-Chuo Medical Center National Hospital Organization, Tsu, Japan; 5 Graduate School of Letters, Kyoto University, Kyoto, Japan; 6 Tezukayama University, Nara, Japan

**Keywords:** social development, theory of mind, cohort study, diverse desire

## Abstract

**Background:**

The purpose of this study was to examine the relations between children’s social competence and initial index of theory of mind at 30 months of age.

**Methods:**

The participants of the study were 322 toddlers and parents/caregivers who were registered with the Japan Science and Technology Agency (JST) project. They completed a five-minute interaction session, which was coded using the Interaction Rating Scale (IRS) as an evidence-based practical index of children’s social competence. In addition, the children were asked to complete a diverse-desire task as a ToM (theory of mind) index.

**Results:**

The results showed that the ToM index was related to the total score and subscales of the IRS, such as Empathy and Emotional regulation.

**Conclusions:**

These findings show that the IRS score was related to ToM task performance at 30 months of age.

## INTRODUCTION

In recent years, the problematic behaviors of school-age children and adolescents, such as bullying, violence, and impulsiveness, are becoming a serious problem in Japan. There is particular concern about the social development of toddlers because it has an influence on later social competence and problem behavior.

Many studies on early child development have discussed children’s problem behaviors and social skills.^1^ Furthermore, the relationship between children’s understanding of human mental states—their ToM (theory of mind)—and early cognitive development has been studied intensively in the last 20 years.^2^^–^^4^ In Japan, Itakura et al^5^ reported positive correlations between ToM performance based on Wellman and Liu’s Scale of the ToM^6^ when children were 30 months old and early social cognitive development at the age of 4 months.

Social development involves diverse aspects and it is important to consider all of these. Therefore, we developed the IRS (Interaction Rating Scale) based on NCAST (Nursing Child Assessment Satellite Training) teaching scales^7^ for Japanese children from the time of birth to 8 years of age. This scale is widely used in both clinical practice and research for assessing the quality of the dyad of caregiver-child interaction. Previously, Anme et al^8^ observed 38 children suffering from development disorders (ADHD (attention-deficit/hyperactivity disorder)/PDD (pervasive developmental disorder)), mental retardation, abuse, or maltreatment and classified their interaction behavior using the IRS classification system. They found that the IRS scores were significantly related to the children’s behavior problems, thereby confirming the reliability and validity of the scale. Furthermore, the IRS scores show highly significant correlations with the NCAST teaching scales (Child items, *r* = .70; Caregiver items, *r* = .98; Total, *r* = .89). In addition, previous studies have indicated that satisfaction with spousal support in child rearing when children were 4 months old is a factor affecting the social development of children at 18 months of age.^9^

In other words, early social cognitive development such as ToM performance and social development is regarded as one of the dimensions of child social development, and it is assessed using the IRS. However, little detailed research has been conducted on social competence and ToM in young children from the longitudinal perspective—an aspect that requires more consideration.

To address this gap in the literature, the present study sought to examine the relation between ToM development, including the understanding of diverse desires, and positive social competence. The IRS was used to assess these dimensions in children who were 30 months of age.

## METHODS

### Participants

The participants of the study were 322 (aged 30 months) dyads of children and their caregivers, who participated in the JST (Japan Science and Technology Agency) project.

In order to comply with the ethical standards laid down by the JST, before conducting the research, the families of all the participants were made to sign informed consent forms and made aware that they had the right to withdraw from the experiment at any time. As the infants were too young to provide informed consent, we carefully explained the purpose, content, and methods of the study to the caregivers and obtained their consent. To maintain confidentiality, personal information about the participants was collected anonymously, and a personal ID system was used to protect this information. Further, all the image data were stored on a password-protected disk; only the researchers who were granted permission by the chairman were allowed access to the data.

This study was approved by the ethics committee of the JST.

### Measures

The IRS. This scale is used to measure the children’s social competence and the caregivers’ child-rearing competence through five-minute observations of caregiver-child interactions. It is suitable for the assessment of interactions between caregivers and children, right from infancy to the age of eight. The scale includes 70 items that are used to obtain a behavioral score and 11 items that are used to obtain an impression score; these items are grouped into ten subscales. Five subscales focus on children’s social competences: (1) Autonomy, (2) Responsiveness, (3) Empathy, (4) Motor regulation, and (5) Emotional regulation. Another five subscales assess the caregivers’ parenting skills: (6) Respect for autonomy development, (7) Respect for responsiveness development, (8) Respect for empathy development, (9) Respect for cognitive development, and (10) Respect for social-emotional development. Further, one item was used to assess the overall impression of synchronous relationships. A training manual on the IRS has been developed for practitioners and researchers. Two different sets of variables are scored: behavior items and impression items for each subscale. Each subscale assesses the presence of behavior (1 = Yes, 0 = No), and the sum of all items in the subscale provides the overall behavior score.

The impression items and overall impression item were rated on a five-point scale, where 1 = not evident at all, 2 = not evident, 3 = neutral, 4 = evident, 5 = high level of evidence. The evaluator completes the checklist composed of 25 items focusing on children’s behavior toward caregivers (eg, child looks at the caregiver’s face as a form of social referencing) and 45 items focusing on caregiver behavior. The observer then provides an impression on a 5-point scale for the level of development for each subscale and for an overall impression.

Diverse desire. We especially paid attention to diverse desire in the ToM. In this study we used a ToM tasks.^10^ These tasks were used to measure the children’s cognitive development through structured investigation for several minutes. This study attempted to facilitate the procedure of the ToM task using a new method involving the display of picture choices on PC monitors instead of puppets version or picture stories version.^5^ Through these tasks, the child understands that two persons (the child vs someone else) make different choices from among the same set of objects.

### Procedure

In this study, the IRS was employed in the following manner: a five-minute video recording of the setting of the child-caregiver interaction (the child and caregiver playing with blocks and putting them in a box) was made. The caregiver-child interactions were videotaped in a controlled laboratory environment. The recording was carried out in a room with five video cameras; one camera was placed at each of the four corners and the fifth was placed in the center of the ceiling. The dyads of children were escorted into a room (with dimensions of 4 × 4 meters) furnished with a small table and a small-sized chair meant for a child. The caregiver introduced herself to the child and interacted with the child in a natural manner, just as she would on a regular day.

In order to score the behavior, two members of the research team coded the behaviors observed. A third child professional, who had no contact with the participants, also scored the behavior. The behavior of the children and caregiver during the caregiver-child interaction was coded as follows. If the child displayed the behavior described in the item, a score of 1 was given; conversely, if the child failed to display the behavior described in the item, a score of 0 was given. A child’s total score was the sum of the score that he/she received on all the subscales. A higher score indicated a higher level of development. The same method of coding was used to evaluate the caregivers’ behavior. The total IRS score was the total score of the child plus the total score of the caregiver.

Diverse-desire task performance was evaluated as follows: The children were tested in the same quiet room with their mothers and the examiner. The diverse-desire task constituted one subset of tasks in which children saw a boy figure, a cookie, and a carrot on the PC monitor at the same time. They answered or chose between one of these pictured choices. The children were led through a series of questions about snacks (eg, Which snacks do you like?) and then shown illustrations of a cookie and a carrot on a PC monitor. After they had answered the questions, the examiner told them that the boy in the PC monitor liked the other choice. The child was then asked about the snack chosen by the boy in the PC monitor (eg, Which snacks does he choose?). There are two components in the diverse-desire task. The first task required the children to state their choice. This question has been referred to as the knowledge of self-desire. The second task asked children to explain which snacks the boy in the PC monitor would choose. The behavior of the children was coded as follows. First of all, if the child did not respond, a score of 0 was given. Second, if the child could state his/her own preference, a score of 1 was given. Third, if the child could distinguish his/her own desire from that of the other, a score of 2 was given.

## RESULTS

Demographic analysis of the families participating in this study (Table 1) revealed that the distribution of boys, 157 (48.8%), and girls, 165 (51.2%), was fairly even. The mothers’ age ranged from 20 to 43 years, with more than half of them (209, 64.9%) between 30 and 39 years of age. The fathers’ age ranged from 20 to 58 years, and their distribution was similar to that of the mothers.

**Table 1.** Demographic Information

Items	*n*	%
Gender		
Boys	157	48.8
Girls	165	51.2
Siblings		
No	171	53.1
Yes	149	46.3
No answer	2	0.6
Family type		
Nuclear family	279	86.6
Extended family	36	11.2
No answer	7	2.2
Mother’s age		
20–29	99	30.7
30–39	209	64.9
40–49	13	4.0
No answer	1	0.3
Father’s age		
20–29	74	23.0
30–39	202	62.7
40–49	27	8.4
50–	4	1.2
No answer	15	4.7
Mother’s career		
No	167	51.9
Yes	155	48.1
Mother’s education		
Middle school	9	2.8
High school	70	21.7
Vocational school	66	20.5
Short-term college	83	25.8
University	86	26.7
Post-college	3	0.9
No answer	5	1.6
Father’s career		
No	1	0.3
Yes	321	99.7
Father’s education		
Middle school	6	1.9
High school	106	32.9
Vocational school	43	13.4
Short-term college	6	1.9
University	119	37.0
Post-college	23	7.1
No answer	19	5.9
Family Income		
<2 million JPY	14	4.3
2–4 million JPY	88	27.3
4–6 million JPY	138	42.9
6–8 million JPY	43	13.4
8–10 million JPY	17	5.3
≧10 million JPY	15	4.7
No answer	7	2.2

Total	322	100.0

JPY, Japanese yen.

The family data showed a relatively broad range of education and income levels: 2.8% of the mothers and 1.9% of the fathers only had a middle school education. The annual family income levels ranged from under 2 million Japanese yen (JPY) (14, 4.3%) to 4 million–6 million JPY (138, 42.9%), the income level that had the highest frequency.

Table 2 shows the frequencies of the diverse-desire task performance: “no reaction,” 8 (2.5%), “states own preference,” 162 (50.3%), and “recognition of diverse desires,” 152 (47.2%).

**Table 2.** The frequencies of the diverse-desire task at 30 months

Category	*n*	%
no reaction	8	2.5
states own preference	162	50.3
recognition of diverse desires	152	47.2

Correlations between the ToM task performance and IRS score (Table 3) revealed that the performance of the diverse-desire task was correlated with the children’s social competence (*r* = 0.15, *P* = 0.009), including Empathy (*r* = 0.12, *P* = 0.032), Motor regulation (*r* = 0.22, *P* < 0.001), and Emotional regulation (*r* = 0.15, *P* = 0.006).

**Table 3.** The relationship between diverse-desire task performance and children’s social competence

Items	30 months					

	Social competence	Autonomy	Responsiveness	Empathy	Motor regulation	Emotional regulation
diverse-desire task	0.15	0.06	0.03	0.12	0.22	0.15
	0.009^b^	0.319	0.634	0.032^a^	<0.001^b^	0.006^b^

upper line: correlation coefficient. lower line: probability value (*P*). ^a^*P* < 0.05. ^b^*P* < 0.01.

## DISCUSSION

This study provides new findings about early social development.

First of all, the present study provides us with a basic understanding of the relations between the cognitive development of toddlers and ToM at 30 months; thus, it provides an interesting insight into people’s mental states. Previous studies have revealed that most of the children performed well on the diverse-desire task at 18-month-old and 3 years of age.^6^^,^^11^ However, in this study, almost half the children who were 30 months of age performed well on the task. This result suggested that this age is one of the important stages in children’s social development and could offer a better understanding of people’s mental states.

Second, in this study, we examined the relations between children’s social competence at 30 months of age by using the IRS and one of the ToM tasks used in cohort studies. The IRS was employed because it can be used with the same subscales framework across a wide range of ages for children—from infants to school-going age.

Third, we found that the IRS score was related to ToM task performance at 30 months of age. The diverse desire in the ToM task needs to control your own desire and choose the correct desire which the other person has. Therefore, this ToM task has a possibility including regulation. This study indicated the correlations between Empathy, Motor regulation, and Emotional regulation (social competences) and the performance of the diverse-desire task, which required the children to recognize another person’s choice when it differed from their own. Human behavior is caused by mental states; this phenomenon has been referred to as “having a theory of mind”.^12^ ToM is assessed through mentalizing tasks in which participants have to understand the behavior of characters in terms of their mental states. These tasks typically involve false beliefs and can be presented as stories or cartoons.^13^ Therefore, ToM is one of the important viewpoints for understanding the cognitive processes and cognitive development of children. One of the most important findings of our study was the relation between cognitive development and social competence, especially empathy and self-regulation, at 30 months of age; this provides an interesting insight into people’s mental states. As per the common-employed framework, motor regulation and emotional regulation clarify self-regulation. Empathy is a psychological construct regulated by both cognitive and affective components, which interact in a systemic manner to produce emotional understanding.^14^^–^^16^ The results of the present study suggest that early social mentalizing is related to other social dimensions at 30 months of age. Thus, it might be possible to gain an understanding of the features of children’s social development by adopting such an approach.

While this study provides valuable findings, it is also important to acknowledge its limitations. First, the study adopted a cross-section approach for children who were 30 months of age. Because continuity is important when we discuss the development of children, further research in this cohort has the potential to reveal the features of early social development. Second, the IRS and ToM tasks might not cover all dimensions of social skills, although we used the most common frameworks of social skills.

In conclusion, this study revealed a correlation between IRS scores and a ToM task at 30 months of age. Follow-up research at 42 months could clarify the features of early social development and provide information that is useful to caregivers and child-care professionals.
